# Navigating the meta-crisis of generativity: adapting qualitative research quality criteria in the era of generative AI

**DOI:** 10.3389/frma.2025.1685968

**Published:** 2025-11-04

**Authors:** Niroj Dahal, Md. Kamrul Hasan, Amine Ounissi, Md. Nurul Haque, Hiralal Kapar

**Affiliations:** ^1^Kathmandu University School of Education, Lalitpur, Nepal; ^2^Department of English, United International University, Dhaka, Bangladesh; ^3^Centre for Foundation and Continuing Education, University Malaysia Terengganu, Kuala Nerus Terengganu, Malaysia; ^4^Department of English and Modern Languages, International University of Business Agriculture and Technology, Dhaka, Bangladesh

**Keywords:** generative AI, qualitative research, crisis of representation, crisis of legitimation, crisis of praxis, algorithmic bias, posthuman ethics

## Abstract

Integrating generative AI (GenAI) in qualitative research offers innovation but intensifies core epistemological, ontological, and ethical challenges. This article conceptualizes the meta-crisis of generativity—a convergence of Denzin and Lincoln's three crises: representation (blurring human/AI authorship), legitimation (questioning trust in AI-generated claims), and praxis (ambiguity in non-human participation). We examine how human-GenAI collaboration challenges researchers' voice, knowledge validity, and ethical agency across research paradigms. To navigate this, we propose strategic approaches: preserving positionality via voice annotation and reflexive bracketing (representation); ensuring trustworthiness through algorithmic audits and adapted validity checklists (legitimation); and redefining agency via participatory transparency and posthuman ethics (praxis). Synthesizing these, we expand qualitative rigor criteria—such as credibility and reflexivity—into collaborative frameworks that emphasize algorithmic accountability. The meta-crisis is thus an invitation to reanimate the critical ethos of qualitative research through interdisciplinary collaboration, balancing the potential of GenAI with ethical accountability while preserving humanistic foundations.

## 1 Introduction

OpenAI introduced ChatGPT in November 2022, a conversational Generative Artificial Intelligence (GenAI) system that offers unrestricted access and advanced language processing capabilities (Dahal, 2024b). Integrating generative artificial intelligence tools in qualitative research has flashed transformative possibilities—and profound ethical, epistemological, and ontological challenges. Building on the foundational “crises” articulated in the early 90s—representation, legitimation, and praxis (Denzin and Lincoln, 2005)—this article introduces the meta-crisis of generativity: a convergence of uncertainties arising when human researchers collaborate with GenAI to produce knowledge.

The advent of Generative Artificial Intelligence (GenAI) tools has brought about a paradigm shift in qualitative research (Agarwal, 2025; Bai and Wang, 2025; Baytas and Ruediger, 2025; Bozkurt et al., 2024; Chan and Hu, 2023; Dahal, 2024b; Haouam, 2025; Hughes et al., 2025; Karataş et al., 2025; Owoahene Acheampong and Nyaaba, 2024; Yildirim et al., 2025; Zawacki-Richter et al., 2019; Zhou et al., 2024). While these tools promise efficiency and enhanced analytical capabilities (Agarwal, 2025; Arosio, 2025; Bai and Wang, 2025; BaiDoo-Anu and Owusu Ansah, 2023; Baytas and Ruediger, 2025; Bennis and Mouwafaq, 2025; Bozkurt et al., 2024; Burleigh and Wilson, 2024; Chan and Hu, 2023; Dahal, 2023, 2024a; Drinkwater Gregg et al., 2025; Fui-Hoon Nah et al., 2023; García-López and Trujillo-Liñán, 2025; Haouam, 2025; Hitch, 2024; Hughes et al., 2025; Ilieva et al., 2025; Jack et al., 2025; Karataş et al., 2025; Lakhe Shrestha et al., 2025; Moura et al., 2025; Nguyen-Trung, 2025; Owoahene Acheampong and Nyaaba, 2024; Sun et al., 2025; Wood and Moss, 2024; Yildirim et al., 2025; Zawacki-Richter et al., 2019; Zhang et al., 2025; Zhou et al., 2024), they raise critical concerns regarding authorship, representation, legitimacy, and praxis. These concerns resonate with the foundational crises conceptualized by Denzin, and Lincoln in the early 90s (Denzin and Lincoln, 2005). In this transformed research climate, we propose that these crises be understood as components of a broader challenge: the meta-crisis of generativity.

Likewise, integrating generative artificial intelligence (GenAI) in qualitative research has created remarkable opportunities for innovation while intensifying foundational epistemological, ontological, and ethical challenges (BaiDoo-Anu and Owusu Ansah, 2023; Bennis and Mouwafaq, 2025; García-López and Trujillo-Liñán, 2025; Hitch, 2024; Ilieva et al., 2025; Moura et al., 2025; Nguyen-Trung, 2025; Sun et al., 2025). This article conceptualizes the meta-crisis of generativity—a convergence of three interconnected crises adapted from the seminal work of Denzin and Lincoln (2005): representation (who speaks?), legitimation (can we trust this knowledge?), and praxis (who participates in change?). By exploring the implications of human-GenAI collaboration, this article examines how qualitative researchers can navigate the diminishing authorial voice, the lack of clarity in AI-generated knowledge claims, and the ethical ambiguities surrounding non-human agency in traditional, modern, and community-oriented qualitative research.

First, we discuss the crisis of representation, which explores the ontological tensions between human subjectivity and GenAI's synthetic objectivity, proposing strategies such as voice annotation and reflexive bracketing to preserve the researcher's positionality. Second, we examine the crisis of legitimation that arises from epistemological uncertainties associated with AI's hidden biases and algorithmic opacity, advocating for algorithmic audits and adapted validity checklists to ensure trustworthiness. Third, the crisis of praxis examines ethical dilemmas in traditional, modern, and community-oriented qualitative research when GenAI tools act as co-participants, urging frameworks for participatory transparency and posthuman ethics that redefine agency. Synthesizing these challenges, the article expands a revised framework for qualitative rigor, reimagining traditional criteria like credibility and reflexivity through the lens of algorithmic accountability and human-AI dialogue.

Thus, we argue that the meta-crisis is not a threat but an invitation to reanimate the critical ethos of qualitative research, demanding interdisciplinary collaboration to balance GenAI's generative potential with ethical accountability. Thus, integrating interpretive, critical, and postmodern paradigms empowers researchers to leverage GenAI as a provocateur while preserving the humanistic foundations of qualitative inquiry. To this end, this article addresses the following questions:

- How can researchers ensure their thoughts and personal views stay clear in their work when AI tools—built on incomplete and biased data—might replace or distort their understanding of people's stories?
- Can we still trust research that uses AI, even though AI systems are often unclear and carry hidden biases? What new rules or checks do we need to implement to ensure that AI-generated insights are fair and reliable?
- If AI is used as a collaborator in research with communities, how do we deal with the fact that it's not human—while still respecting the community's right to make decisions, take responsibility, and push for empower or change?

## 2 Literature review

A review of existing literature on the meta-crisis of generativity while adapting qualitative research quality criteria in the era of Generative AI was conducted to contextualize the authors' reflections and provide supporting evidence.

## 3 Methodology

This perspective article is based on the authors' personal insights and opinions, drawing from their experiences on the meta-crisis of generativity while adapting qualitative research quality criteria in the era of Generative AI.

### 3.1 Data sources

The authors' personal experiences and observations, along with relevant literature, are the foundation of this article.

## 4 Meta-crisis of generativity

The meta-crisis of generativity underscores the profound disruption of qualitative research principles brought about by the integration of Generative AI (GenAI) in knowledge production Dahal, (2024a); García-López and Trujillo-Liñán, (2025); Moura et al., (2025). This crisis unfolds across three interrelated dimensions: representation (ontology), legitimation (epistemology), and praxis (ethics). The crisis of representation questions how to distinguish between human and AI voices in co-authored texts and how to authentically convey lived experiences when GenAI-generated content—shaped by biased and fragmented datasets—mimics objectivity, potentially silencing the researcher's reflexive voice. The crisis of legitimation challenges the trustworthiness of AI-assisted research, as opaque algorithms and biased training data undermine traditional standards of credibility and transferability. Likewise, the crisis of praxis interrogates the ethical implications of AI acting as a participant or agent in change-oriented research, such as autoethnography or Participatory Action Research (PAR), raising concerns about power imbalances and accountability. Understanding these dimensions is essential for adapting qualitative methodologies to responsibly integrate GenAI, which requires methodological innovation and reflexivity to uphold research integrity.

### 4.1 Crisis of representation: “Who speaks?”—ontological challenges

The crisis of representation asks: How do we authentically portray lived experiences when the researcher's voice is entangled with GenAI's synthetic text? In traditional qualitative research, the researcher's subjectivity is foregrounded; GenAI disrupts this by introducing an “objective” synthetic voice trained on biased, fragmented datasets. One of the most significant challenges in qualitative research involving GenAI is the blurred line between content generated by AI and that produced by human researchers. However, qualitative research is grounded in subjective interpretations, which reflect the unique perspectives and insights of human researchers. However, AI-generated outputs often present information in a seemingly objective manner, making it difficult to attribute specific parts of the research to either the AI or the human contributors. This indistinct boundary complicates the process of determining authorship and understanding the nuances of the research findings.

#### 4.1.1 GenAI's impact on ontology

GenAI tools like ChatGPT generate text that lacks embodied experience, cultural context, and emotional resonance. This leads to a loss of human nuance in research outputs. Additionally, when AI drafts field notes or analyzes data, the human researcher's reflexive voice risks being diluted, potentially leading to the erasure of positionality, a crucial element in qualitative research. Case studies have demonstrated this problem. For example, in a hybrid autoethnography on migrant labor where ChatGPT drafted interview summaries, cultural nuances were inadvertently flattened. Similarly, in a collaborative study on mental health, AI-generated themes conflicted with participants' lived realities, which underline the ontological challenges of integrating GenAI in qualitative research.

#### 4.1.2 Strategies for clarifying representation

To ensure transparency in GenAI-assisted qualitative research, researchers can implement several integrated strategies. First, explicitly document all AI involvement in methodology sections—specifying roles in data collection, analysis, or writing—using disclosure statements. Second, employ voice labeling with typographic cues (e.g., AI-generated text vs. human reflections) to distinguish authorship. Third, practice reflexive bracketing: position GenAI as a “provocateur” for initial drafts, then critically interrogate its outputs through iterative human-AI dialogue to surface biases. Fourth, formalize co-authorship frameworks that ethically credit GenAI contributions (e.g., acknowledging tools like GPT-4 as “non-human collaborators”). Finally, a reflective journal should be maintained to track AI interactions and content modifications, supplemented by peer debriefing to assess the alignment between human interpretation and AI-generated content. Thus, these approaches preserve research integrity while leveraging GenAI's analytical capabilities.

### 4.2 Crisis of legitimation: “Can we trust this knowledge?”—epistemological challenges

The Crisis of Legitimation centers on epistemological concerns, specifically how we can validate the knowledge claims produced through AI-human collaborations. AI tools are trained on extensive datasets that are inherently biased, which raises significant questions about the validity and reliability of the findings generated with AI assistance. This issue is crucial for ensuring that the knowledge produced is credible and trustworthy. Legitimation concerns the trustworthiness of knowledge claims. GenAI's reliance on biased training data (e.g., excluding marginalized voices) and opaque algorithms undermines traditional validity criteria, such as credibility and transferability.

#### 4.2.1 GenAI's impact on epistemology

GenAI tools reproduce structural inequalities embedded in their training corpora (e.g., racial, gender, or cultural biases). This leads to hidden biases in AI-generated analyses. Additionally, researchers cannot fully trace how AI tools code data or generate themes, violating interpretive transparency. This “black box” nature of AI analysis challenges the epistemological foundations of qualitative research. For instance, a GenAI system analyzing marginalized communities may lack cultural nuance due to limitations in its training data. This raises important questions about the epistemological validity of AI-assisted qualitative research.

#### 4.2.2 Strategies for strengthening trustworthiness

To safeguard the validity of AI-assisted qualitative research, researchers should implement multifaceted strategies: (1) Conduct algorithmic audits with data scientists to document training data sources, decision pathways, and analytical roles; (2) Employ triangulation by cross-verifying AI outputs with independent human coding, multi-tool comparisons, and traditional techniques like member-checking and thick description; (3) Maintain rigorous audit trails logging all AI interactions, prompt iterations, and textual modifications; (4) Embed bias mitigation through critical interrogation of AI assumptions and counter-frameworks that challenge dominant discourses; and (5) Adapt validity checklists (e.g., Lincoln and Guba, 1985) to include AI transparency metrics and protocols like Trustworthiness Audits. These measures uphold epistemological integrity while harnessing the analytical capabilities of GenAI.

### 4.3 Crisis of praxis: “Who participates in change?”—ethical challenges

In methodologies such as autoethnography and Participatory Action Research (PAR), researchers actively engage as participants in the research process. This involvement prompts a provocative question: should AI tools also be recognized as co-participants in the design, execution, and reporting of research? This consideration is crucial as it challenges traditional notions of participation and acknowledges the significant role AI can play in shaping research outcomes. Participatory action research (PAR) prioritizes co-creation with communities to drive social change. But when GenAI tools act as “participants” (e.g., designing surveys, analyzing narratives), their role as non-human agents raises ethical dilemmas.

#### 4.3.1 GenAI's impact on praxis

When GenAI tools are used in PAR, such tools create power imbalances, as communities may perceive AI as an extension of institutional authority. Additionally, there is an agency ambiguity: can an AI tool be a “change agent” if it lacks intentionality? For example, in a PAR project on school transformation projects where ChatGPT was used to analyze stakeholder interviews, community members expressed distrust, perceiving AI as an extension of institutional authority rather than a neutral analytical tool. This underlines the ethical complications of integrating GenAI into participatory research methodologies.

#### 4.3.2 Strategies for ethical integration of AI in research praxis

To navigate the Crisis of Praxis, researchers must ethically reconceptualize GenAI's role by acknowledging its agency as a co-constructor of knowledge rather than a neutral tool. Drawing on actor-network theory (Latour, 2005) and posthuman ethics, this involves: (1) collaborative boundary-setting with communities (e.g., limiting AI to “scribe” roles to mitigate power imbalances); (2) participatory transparency through plain-language disclosure in consent processes and accountability statements; and (3) critical reflexivity via ethical guidelines that uphold autonomy and informed consent. These approaches reframe non-human participation while preserving the humanistic foundations of qualitative research.

## 5 Adapting quality criteria for the GenAI era: toward a new framework

The Meta-Crisis of Generativity demands a paradigm shift in how we define quality in qualitative research. Traditional criteria for evaluating qualitative research, as established by Lincoln and Guba, 1985, need to be reimagined to account for the integration of GenAI tools.

### 5.1 Contemporary paradigms

Interpretivism, criticalism, and postmodernism research paradigms demand adapted quality criteria that balance human subjectivity with GenAI's generative potential. Within interpretivism, AI can identify latent themes in data; however, researchers should manually contextualize cultural nuances by revising AI-generated thematic maps through member checking to ensure alignment with participants' lived experiences (Combrinck, 2024). Criticalism leverages GenAI to expose power structures (e.g., detecting gender bias in corporate documents), though such insights require validation by marginalized stakeholders to confirm inequities. Postmodernism uniquely embraces GenAI's capacity to destabilize singular “truths,” employing it to generate contradictory narratives for ideological deconstruction—such as producing competing interpretations of social events to reveal underlying tensions.

### 5.2 A new framework for qualitative rigor

We propose a new framework for evaluating quality in GenAI-assisted qualitative research based on these interpretivism, criticalism, and postmodernism research paradigms. Table 1 shows the traditional and GenAI-adjusted criteria.

**Table 1:** Traditional and GenAI-adjusted criterion.

**Traditional criteria**	**GenAI-adjusted criteria**
Credibility	Algorithmic accountability
Transferability	Contextual embedding logs
Dependability	System transparency documentation
Confirmability	Human-AI dialogue journaling
Reflexivity	AI-Human co-reflection records

To maintain research rigor while leveraging GenAI, qualitative researchers should adopt three interconnected principles: generative transparency through documenting all human-AI interactions in research logs by researchers for ensuring the ethical relationality by prioritizing human accountability over AI efficiency to ensure ethical compliance; and postmodern pluralism that embraces GenAI as a critical “provocateur” challenging human-centric epistemology (BaiDoo-Anu and Owusu Ansah, 2023; Burleigh and Wilson, 2024; Dahal, 2023; Haouam, 2025; Zhang et al., 2025). Thus, these adapted criteria enable researchers to harness GenAI's potential while safeguarding the integrity essential to qualitative inquiry.

## 6 Discussion

The integration of Generative AI (GenAI) in qualitative research has triggered what can be described as a meta-crisis of generativity—an entanglement of Denzin and Lincoln's (1994) foundational crises of representation, legitimation, and praxis (Agarwal, 2025; Bai and Wang, 2025; Baytas and Ruediger, 2025; Haouam, 2025; Ilieva et al., 2025; Yildirim et al., 2025). This convergence challenges the epistemological and ethical foundations of qualitative inquiry. GenAI's so-called “synthetic objectivity” often masks the researcher's subjectivity, diluting positionality and flattening cultural nuance, particularly when AI is used to generate field notes or thematic codes. This is evident in cases like autoethnographies of migrant labor and/or STEAM teachers, where lived experiences risk being erased. Such representational issues directly contribute to legitimation crises, as the opaque nature of AI algorithms and their biased training data undermine trustworthiness, thereby violating credibility standards, such as those proposed by Lincoln and Guba (1985). In participatory research contexts, such as Participatory Action Research (PAR), GenAI's role as a “co-participant” introduces power dynamics, with communities potentially perceiving AI as a proxy for institutional authority, thereby destabilizing the ethics of co-creation. These challenges necessitate integrated strategies to maintain qualitative rigor (Dahal, 2023). For representation, techniques like voice annotation and reflexive bracketing can foreground human voices, while co-authorship frameworks that credit AI (e.g., GPT-4) as a non-human collaborator help mitigate ontological erasure. To address legitimation, algorithmic audits and triangulation practices enhance transparency, while member-checking, alongside thick description, anchors AI outputs in human interpretation. In terms of praxis, participatory transparency—such as limiting AI to scribe roles with community consent—helps strike a balance between efficiency and ethical relationality. Rigor itself must be reimagined: algorithmic accountability can replace traditional credibility by documenting training data biases, while Human-AI dialogue journaling offers a new form of confirmability by tracing how human reflexivity shapes AI outputs. Postmodern pluralism, meanwhile, leverages GenAI to challenge hegemonic narratives and foster counter-narratives (Haouam, 2025). Rather than signaling an endpoint, this meta-crisis invites a reanimation of the critical ethos of qualitative research. It calls for interdisciplinary collaboration among data scientists, ethicists, and communities to co-design culturally attuned AI tools for Global East-West contexts. Actor-network theory (Latour, 2005) offers a lens for reconceptualizing AI as an actor within knowledge networks, thereby demanding ethical frameworks for non-human agency. Ultimately, paradigmatic synergy—drawing on interpretivist, critical, and postmodern traditions—can resist techno-determinism and center the voices of marginalized individuals. Yet, tensions remain unresolved: Can AI ever embody phronesis, or practical wisdom, in participatory change? The field urgently needs standardized disclosure protocols for AI use, decolonized training datasets to prevent epistemic violence, and longitudinal studies to assess the long-term impact of GenAI on research praxis.

## 7 Conclusion

As qualitative research adapts to the integration of GenAI tools, the challenges of authorship, trustworthiness, and ethical praxis require proactive strategies to maintain research integrity. The Meta-Crisis of Generativity—comprising the Crisis of Representation, the Crisis of Legitimation, and the Crisis of Praxis—demands methodological innovation and reflexivity. The Meta-Crisis of Generativity is not a threat, but an invitation to reanimate the critical spirit of qualitative research. Researchers can harness GenAI's potential while safeguarding ethical rigor by adapting Denzin and Lincoln's different forms of crises and representations (Denzin and Lincoln, 2005). This requires interdisciplinary collaboration among ethicists, communities, and engineers to forge tools and norms that honor the humanistic roots of qualitative research. Thus, qualitative researchers can navigate this evolving landscape while upholding the foundational values of rigor, trust, and ethical responsibility by transparently documenting AI's involvement, critically assessing claims, and ethically integrating AI in research praxis. Thus, future discussions should focus on establishing best practices for AI-human collaboration in qualitative research, developing ethical guidelines, and refining methodologies to align with contemporary interpretivist, criticalist, and postmodernist paradigms. As the field evolves, qualitative researchers must engage in continuous dialogue to ensure that AI serves as a tool for enhancement rather than a disruptor of research integrity.

## 8 Limitations

This article's limitations include:

- Acknowledges that the subject matter is purely conceptual in nature.
- Subjective nature of personal insights and opinions.
- Limited generalizability due to focus on experiences on the meta-crisis of generativity while adapting qualitative research quality criteria in the era of Generative AI.

## 9 Recommendations

The recommendations of the article include:

- Label AI-generated content clearly and use GenAI as a draft initiator, followed by human critique to retain positionality and nuance.
- Audit AI training data and triangulate outputs with human validation methods, such as member-checking.
- Disclose AI's role, obtain consent, and set boundaries—e.g., limiting AI to support roles in participatory research.
- Utilize GenAI-specific criteria, such as algorithmic accountability and dialogue journals, to replace traditional validity criteria.
- Partner with ethicists, engineers, and communities to build culturally sensitive AI tools and ethical standards.

## Data Availability

The original contributions presented in the study are included in the article/supplementary material, further inquiries can be directed to the corresponding author.
